# Characterization and Genetic Evolution of H6N2 Subtype AIV Isolates from Aquatic Birds

**DOI:** 10.3390/microorganisms14040895

**Published:** 2026-04-16

**Authors:** Lulu Deng, Taif Shah, Yandaijiu Wang, Peng Cheng, Yushan Kui, Binghui Wang, Xueshan Xia

**Affiliations:** 1 Faculty of Life Science and Technology, Kunming University of Science and Technology, Kunming 650500, China; 3055779157@aliyun.com (L.D.); kuiyushan@aliyun.com (Y.K.); 2 Yunnan Province Key Laboratory of Public Health and Biosafety, School of Public Health, Kunming Medical University, Kunming 650500, China; taifshah@yahoo.com (T.S.); 18313809772@163.com (Y.W.); cheng20250226@163.com (P.C.)

**Keywords:** avian influenza viruses, birds, genetic evolution, growth kinetics, BALB/c mice model

## Abstract

Birds serve as the primary natural reservoirs for avian influenza viruses (AIVs), harboring nearly all known AIV subtypes. The seasonal migratory movements of wild birds play a significant role in the transmission and dissemination of AIVs. Jianhu Lake in Dali, Yunnan Province, serves as a vital congregation point along avian migratory routes, providing an ideal habitat for birds. In this study, a total of 619 avian samples were collected from the Jianhu area, from which four H6N2 subtype AIV strains were successfully isolated. Among these, A/grey heron/Jianhu/JH-89/2024 (hereafter referred to as JH-89) and A/grey heron/Jianhu/JH-91/2024 (JH-91) were isolated from grey herons (*Ardea cinerea*); A/mareca penelope/Jianhu/JH-2-11/2025 (JH-2-11) from a Eurasian wigeon (*Mareca penelope*); and A/duck/Jianhu/JH-1-1/2025 (JH-1-1) from a domestic duck (*Anas platyrhynchos domesticus*). Genomic analyses revealed that these four H6N2 isolates belong to the Eurasian lineage, with all eight gene segments originating from complex reassortment events among diverse Asian isolates. In vitro assays demonstrated that the representative strain JH-2-11 replicated efficiently in various human- and animal-derived cell lines. In vivo infection models revealed that, without prior adaptation, the JH-2-11 strain successfully infected BALB/c mice, resulting in suppressed body weight gain and severe pathological lesions in the respiratory tract (nasal turbinates, trachea, and lungs), without causing mortality or extrapulmonary dissemination. Collectively, although these H6N2 viruses evolve primarily within avian hosts, they exhibit potential for mammalian adaptation and require continuous epidemiological monitoring.

## 1. Introduction

Influenza A viruses (IAVs) belong to the Orthomyxo-viridae family and contain eight segments of negative-sense single-stranded RNA [1]. AIVs can infect both humans and animals and are classified as highly pathogenic (HPAIVs) or low pathogenic (LPAIVs) based on their pathogenicity in chickens [2]. Influenza A viruses have eight negative-stranded RNA segments (PB2, PB1, PA, HA, NP, NA, M, and NS), which are further classified into 18 hemagglutinin (HA) and 11 neuraminidase (NA) subtypes based on their surface glycoproteins [2]. H6 is a widely distributed AIV subtype with low pathogenicity in birds and a diverse range of hosts, including wild aquatic birds, domestic poultry, and mammals [3]. The pathological changes caused by H6N2 in early AIV-infected chickens are primarily linked to mild respiratory infections and yolk peritonitis [4].

While typically classified as low pathogenic (LPAI) in poultry, H6 viruses serve as a vital genetic reservoir, frequently providing internal gene modules for highly pathogenic AIVs, such as H5N1 and H5N6 [3], In China, H6 AIVs have become enzootic in domestic poultry since 2000, with a notable increase in prevalence across southern regions [5,6]. The public health significance of this subtype was underscored by the first human H6N1 infection in Taiwan in 2013 and the subsequent isolation of avian-origin H6N6 from swine herds in Guangdong [7,8]. Recent evidence suggests that several H6 lineages have naturally acquired the ability to bind human-type α-2,6 sialic acid receptors, facilitating their replication in mammalian respiratory models [9,10].

The adaptation of AIVs to mammalian hosts is a multi-genic process governed by specific molecular markers. Key mutations in the hemagglutinin (HA) protein, such as Q226L and G228S, are well known to shift receptor preference from avian-type (α-2,3) to human-type (α-2,6) [11,12]. Furthermore, substitutions in the polymerase basic protein 2 (PB2), notably E627K and D701N, significantly enhance viral replication at the lower temperatures of the mammalian upper respiratory tract [13]. While many circulating H6 strains lack these canonical markers, alternative mutations in the PA, NS1, and M segments have been shown to compensate for such deficiencies, potentially increasing virulence through unique reassortment patterns [14,15]. Consequently, continuous genomic surveillance is essential to track the emergence of these non-canonical adaptive markers.

Despite extensive surveillance in coastal provinces such as Guangdong and Shanghai [6,16,17]. The molecular epidemiology of H6 AIVs in Southwest China remains under-characterized. Yunnan Province represents a critical ecological niche, serving as a convergence point for the East Asian-Australasian and Central Asian migratory flyways. This geographic characteristic provides a unique interface for genetic exchange between migratory waterfowl and domestic poultry, yet reports on the pathogenicity of Yunnan-origin H6 isolates in mammalian models are infrequent.

In this study, we characterized four H6N2 AIVs (JH-89, JH-91, JH-1-1, and JH-2-11) isolated from wild and domestic bird feces in Yunnan between 2024 and 2025. Through whole-genome sequencing and phylogenetic analysis, we identified a distinct reassortment pattern in the JH-2-11 isolate, involving its M and NS segments. We further evaluated the replication kinetics of JH-2-11 in multiple human and animal cell lines and its pathogenicity in a mouse model. By comparing these isolates with previously described H6N2 strains, this study aims to elucidate the unique evolutionary trajectory of H6 AIVs in Southwest China and assess their biological potential for cross-species infection.

## 2. Materials and Methods

### 2.1. Experimental Sites and Virus Isolation

This study was conducted at the Jianhu Wetland Provincial Nature Reserve, located in Jianchuan County, Dali Bai Autonomous Prefecture, Yunnan Province, China (approximate altitude 2180 m). As a typical wetland on the Yunnan-Guizhou Plateau, Jianhu Lake serves as a critical ecological hub and a major stopover and wintering ground for migratory birds along the East Asian-Australasian and Central Asian flyways. According to local ecological monitoring, the wetland hosts nearly 30,000 wintering migratory birds annually. The dominant avian populations include various species of herons (*Ardeidae*) and ducks (*Anatidae*), alongside the largest population of purple swamphens (*Porphyrio poliocephalus*) in China, providing an ideal ecological interface for avian influenza virus surveillance. To ensure sample freshness, fresh fecal samples (deposited within 2 h) were collected using sterile swabs immediately after the bird flocks vacated the area. The samples were subsequently placed in viral transport medium. The collected avian fecal samples were subjected to RNA extraction and reverse transcription, followed by DNA barcoding of the mitochondrial cytochrome c oxidase subunit I (COI) gene to confirm host identity. This study was conducted from October 2024 to March 2025 in the migratory bird distribution areas of the Jianhu region in Dali, Yunnan Province, China. A total of 619 avian fecal samples were collected in the Jianhu area, and avian influenza virus screening was performed on all samples, resulting in the detection of 15 positive cases, among which four were identified as H6N2 subtype avian influenza viruses (the corresponding sample metadata are provided in Supplementary Table S1).

**Figure 1:**
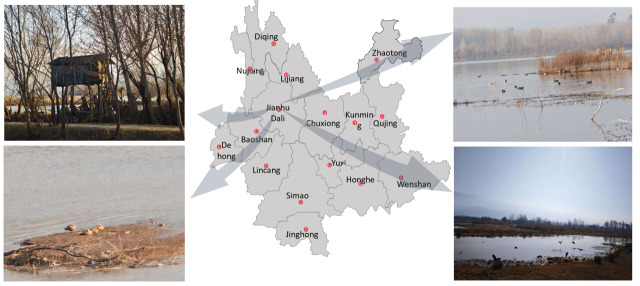
Map showing the sample isolation sites. Jianhu Lake, Dali Bai Autonomous Prefecture.

The JH-89 and JH-91 AIV isolates were isolated from the feces of herons collected from Jianhu Lake in Dali Bai Autonomous Prefecture, Yunnan Province, China, in 2024. In addition, the JH-2-11 and JH-1-1 AIV strains were isolated from the feces of red-necked and domestic ducks in the same lake in 2025 (Figure 1). All AIV strains were isolated and purified by inoculating chicken embryos, and the allantoic fluid was aliquoted and stored at −80 °C for long-term preservation. We used 10-day-old specific pathogen-free (SPF) chicken embryos (Poultry Egg Co., Xinxing Dahua Agri., Co., Ltd., Guangzhou, Guangdong, China) to determine the infection dose for chicken embryos.

### 2.2. Whole Genome Sequencing of AIVs

Viral RNA from the four H6N2 isolates was extracted using the Tiangen Viral RNA Extraction Kit (Tiangen, Beijing, China). Primers were designed using Oligo 7 to amplify the full-length genome of the successfully isolated avian influenza virus, and a phylogenetic tree was constructed based on the full-length sequences using MEGA-X v10.2.6 software (detailed primer information is shown in Supplementary Table S3). The RNA was reverse-transcribed into cDNA using the PrimeScript^TM^ II 1st Stand cDNA Synthesis (Takara, Tokyo, Japan), according to the manufacturer’s guidelines. Using cDNA as a template, each target gene segment was amplified by PCR using the designed primers and Hoffmann et al.’s [18] avian influenza universal primers. The size of the PCR-amplified products was verified on a 1% agarose gel (Vazyme, Nanjing, China). After verification, the samples were subjected to sanger sequencing using an ABI 3730 sequencer (Qingke, Beijing, China).

### 2.3. Genetic Evolutionary Analysis

Sanger sequencing reads were assembled using the Seqman tool in the DNAStar software package (17.3). BLAST comparisons were conducted using the NCBI database to analyze the most similar strains of each gene (amino acid). MEGA-12 software was used with the maximum-likelihood method to construct phylogenetic trees based on the HA, NA, PB2, PB1, PA, NP, M, and NS genetic segments (1000 replications). BioEdit software (7.0.9.0) was used to analyze mutations at key amino acid sites in HA, NA, PB2, PB1, PA, NP, M1, M2, and NS1.

### 2.4. Growth Kinetics of the AIV in Human and Non-Human Cell Lines

The JH-2-11 isolate was specifically selected as the representative strain for subsequent in vitro and in vivo evaluations due to its superior replication fitness (yielding the highest HA and infectious titers among the isolates (related data are shown in Supplementary Table S2)) and its distinct internal gene reassortment pattern involving the M and NS segments. Utilizing this highly fit reassortant ensures robust viral replication in mammalian models, thereby providing a more accurate assessment of the cross-species adaptive potential of currently circulating H6N2 viruses. Briefly, diverse cell lines, including MDCK (Madin-Darby Canine Kidney), VERO (African green monkey kidney), DF-1 (chicken fibroblast), HBE (Human Bronchial Epithelial cells), HEK-293T (Human Embryonic Kidney), and A549 (Human lung adenocarcinoma), were infected at a multiplicity of infection (MOI) of 0.01. The infected cell lines were cultured at 37 °C and 5% CO_2_ for 1–2 h. Following adsorption, the virus-containing solution was withdrawn, and the infected cell lines were washed three times in sterile PBS. After discarding the final wash of PBS, virus growth medium (serum-free, 1% penicillin-streptomycin and cell-line-specific medium supplemented with 2 μg/mL TPCK-trypsin) was added. After 24, 48, 72, 96, and 120 h post-infection (hpi), cytopathic effects were observed in JH-2-11-infected cells, and 200 μL of cell culture supernatant was taken from each well. The experiment was carried out in triplicate. Using MDCK cell lines, the collected supernatants at five-time intervals were utilized to determine the TCID_50_, which was then computed using the Reed-Muench method. Growth curves for the virus in various cell lines were plotted.

### 2.5. Determination of the Chicken Embryo Infectious Dose (EID_50_) of the AIV

The purified virus was serially diluted 10-fold with PBS (10^−4^ to 10^−10^), and each dilution was inoculated into five 10-day-old SPF chicken embryos. (Specific pathogen-free chicken embryos were purchased from Xinxing Dahuanong Poultry & Egg Co., Ltd., Guangzhou, China). The chicken embryos were then incubated at 37 °C for 48 h, and the number of hemagglutination-positive chicken embryos at each dilution was recorded. The EID_50_ of the JH-2-11 AIV strain was calculated using the Reed-Muench method.

### 2.6. Mouse Infection Experiment

Eight 5-week-old female BALB/c mice purchased from Sibeifu Company (Sibeifu, Bio., Co., Ltd., Beijing, China) were intranasally infected with 10^6^ EID_50_/50 μL of virus (50 μL/mouse) [19]. Mice were deeply anesthetized with CO_2_ prior to intranasal inoculation, ensuring the complete inhalation of the viral suspension into the lungs.

Additionally, eight mice were intranasally inoculated with 50 μL of 1× PBS as a negative control. The mice were observed daily, and their body weight changes and mortality were recorded. The baseline weight of the female BALB/c mice on day 0 prior to inoculation was approximately 17.5–18.0 g. The body weights of the mice were monitored and recorded daily (every 24 h) for 12 days post-infection. On the third day of virus inoculation, three mice from each of the negative control and infection groups were randomly euthanized and dissected. Body parts, including the nasal turbinate, intestine, spleen, lung, kidney, and brain of each mouse, were aseptically collected and placed in 1 mL of antibiotic-containing PBS. Each tissue homogenate was centrifuged, and the resulting supernatant was used to determine viral titer by inoculating 10-day-old embryonated chicken eggs. The remaining mice were continuously observed and weighed until the conclusion of the 12th day.

### 2.7. Hematoxylin and Eosin (H&E) Staining

The nasal turbinate and lung tissues of the JH-2-11 infected BALB/c mice were removed and fixed in 4% paraformaldehyde for 48 h, dried through ethanol, embedded in paraffin, and sectioned into thin slices (4 μm). The thin tissue slices were dewaxed with xylene, rehydrated with ethanol, and then stained with H&E. Pathological changes in the nasal turbinate and lung tissues were observed under a microscope and photographed.

### 2.8. Immunohistochemistry (IHC) Staining

The nasal turbinate and lung tissue sections were deparaffinized in xylene and rehydrated through graded ethanol, followed by antigen retrieval and serum blocking. Subsequently, the sections were sequentially incubated with a specific primary antibody (anti-NP antibody; GeneTex, Irvine, CA, USA) and a horseradish peroxidase-conjugated secondary antibody. Finally, the sections were developed using a DAB substrate and counterstained with hematoxylin. The distribution and expression of viral antigens in the nasal turbinates and lung tissues were observed and photographed under a microscope.

### 2.9. Statistical Analysis

All experiments were performed independently in triplicate. Data are presented as the mean ± standard deviation (SD). Statistical analyses and data visualization were conducted using GraphPad Prism software (version 10.1.2).

For cell proliferation kinetics and mouse body weight changes, statistical evaluations were performed using two-way analysis of variance (ANOVA) followed by Tukey’s multiple comparison post hoc test. Differences in viral titers among various tissues were compared using one-way ANOVA. For comparisons between two groups at specific time points, Student’s *t*-test was employed for normally distributed data with equal variance; otherwise, the non-parametric Mann–Whitney U test was applied. Samples with viral titers below the limit of detection (LOD, indicated by dashed lines in the figures) were assigned a value of 0.5 LOD for statistical purposes. *p*-value < 0.05 was considered statistically significant.

## 3. Results

### 3.1. Homology Analysis Revealed Diverse AIV Genetic Origins

The nucleotide sequences of each AIV gene segment were subjected to NCBI BLAST analysis to determine the virus homology (Table 1).

The HA gene sequences of the four newly identified AIVs exhibited the highest homology with that of the H6N2 isolate, A/duck/Kaohsiung/19WB0234-29/2019(H6N2). The NA gene showed sequence homology of 98.30% with A/duck/Bangladesh/58751/2023(H6N2). Among the eight internal genetic segments, the NP segment of the four AIV isolates showed 99.20% sequence similarity to the mallard duck-derived H10N7 in Bangladesh. The PB2 segment sequences showed 99.38% sequence homology with H3N8 subtypes. The PB1 and PA segment sequences exhibited high homology with those of the H4N6 AIV subtype derived from wild and spot-billed ducks in China and South Korea. Strains JH-89, JH-91, and JH-1-1 shared identical top-hit reference strains across all eight gene segments, demonstrating a high degree of genetic uniformity. In contrast, although strain JH-2-11 shared identical origins with the aforementioned three strains across six segments (PB2, PB1, PA, HA, NP, and NA), its M and NS genes exhibited the highest homology with an H10N3 strain from Mongolia, respectively. These homology results further corroborate that strain JH-2-11 possesses independent reassortment origins for its internal genes.

### 3.2. Evolution and Protein Glycosylation Sites in HA and NA

**Figure 2:**
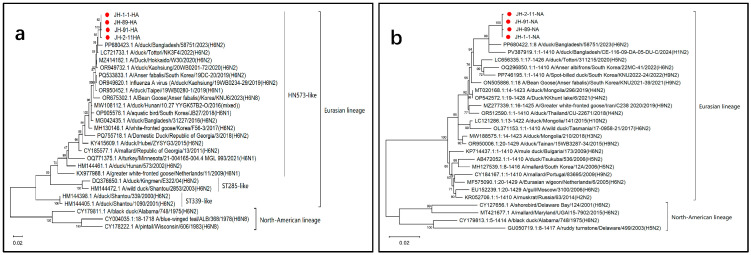
Phylogenetic analysis of four recently discovered H6N2 isolates. Phylogenetic trees based on (**a**) HA and (**b**) NA gene sequences revealed that these viruses are closely related to previously reported AIVs from various countries.

The open reading frame (ORF) of the HA gene sequence was 1701 bp, encoding 566 amino acids. Analysis of the HA gene evolutionary tree revealed that the HA genes of these AIV strains belonged to the Eurasian lineage (Figure 2a). In addition, the ORF of the NA viral gene was 1410 bp, encoding 469 amino acids. According to the evolutionary tree, the NA gene sequences of these four AIV isolates were clustered in the Eurasian branch (Figure 2b). The four AIV strains identified in this study (shown with red dots) clustered closely with H6N2 (GenBank ID: PP680422.1) and H1N2 (GenBank ID: PV387919.1), which were recently isolated from ducks in Bangladesh. This cluster was further embedded in a larger Eurasian lineage that included various NA subtypes (such as H4N2, H6N2, and H10N2), revealing the N2 gene’s widespread circulation and reassortment among different AIV subtypes from other countries. Notably, the H9N2 viruses that clustered closely with our identified subtypes were recently reported (2021–2023), implying that the N2 gene of these H6N2 isolates may have originated from a common ancestor circulating in Asian domestic and wild birds.

The HA gene cleavage site PQIENR↓GLF matches the LPAIV subtypes, which have six potential glycosylation sites: 27NST, 39NVT, 182NNT, 306NKT, 489NGT, and 557NGS (Table 2).

Analysis of key amino acid sites in the HA surface glycoproteins that affect the virus’s receptor-binding characteristics revealed that glutamine (Q) is located at the 226th position and glycine (G) is located at the 228th position, indicating that this virus can bind to avian-type receptors. The detected H6N2 isolates were grouped into the Eurasian HN573-like branch of the phylogenetic tree. These viruses are similar to the H6N2 isolates found in East and Southeast Asian nations such as Bangladesh, Japan, South Korea, Taiwan, and China. Notably, this evolutionary cluster also includes viruses from other NA subtypes, such as H6N1 and H6N8, indicating that the H6 HA gene sequence can reassortment with different NA genes, displaying the subtype’s genetic diversity and evolutionary potential in wild bird populations. Moreover, the NA protein of these AIVs has five potential glycosylation sites: 61NIT, 69NNT, 86NWS, 146NGT, and 234NGT. No resistance mutations, such as E119G, Q136L, or R292K, were identified in the NA protein, indicating that the virus had not developed resistance to NA inhibitors, including zanamivir.

### 3.3. Phylogenetic Analysis Based on the Six Internal Genetic Segments

**Figure 3:**
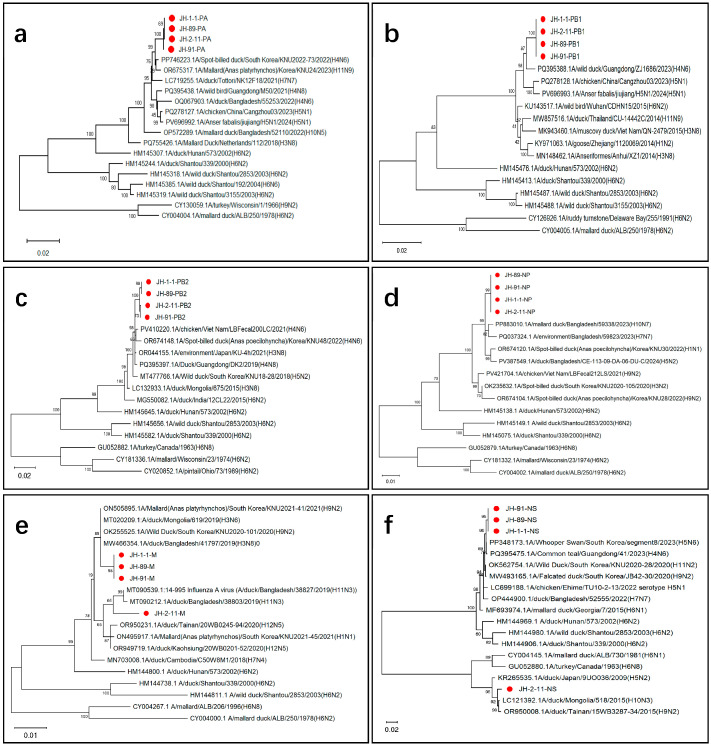
Phylogenetic analysis of the six internal genetic segments showed that H6N2 AIV isolates JH-89, JH-91, and JH-1-1 belonged to the Eurasian lineage. Specifically, the polymerase complex genetic segments (**a**) PA, (**b**) PB1, and (**c**) PB2, as well as (**d**) NP segments of each AIV strain, were clustered in separate clades. In contrast, the evolutionary paths of the (**e**) M and (**f**) NS genetic segments were diverse. The red dots in each phylogenetic tree indicate the four newly identified H6N2 AIV isolates in this study.

To further elucidate the origin and evolutionary characteristics of the internal genes of the four H6N2 avian influenza isolates, phylogenetic trees were constructed for the six internal gene segments (PA, PB1, PB2, NP, M, and NS) (Figure 3). Phylogenetic analyses revealed that all internal genes of these isolates belong to the Eurasian lineage, yet they exhibit highly complex, inter-subtype reassortment signatures. Notably, the evolutionary topologies of these strains varied significantly across different gene segments. In the phylogenetic trees for the ribonucleoprotein (RNP) complex genes (PA, PB1, PB2, and NP), all four isolates (JH-2-11, JH-1-1, JH-89, and JH-91) clustered tightly into an independent monophyletic clade with very high bootstrap support. They shared close homology with H4N6 and H5N1 isolates recently circulating among wild birds and poultry in East and South Asia (e.g., China, South Korea, Vietnam, and Bangladesh), indicating a common evolutionary origin for these four segments in the region.

In Contrast, the phylogenies of the matrix (M) and non-structural (NS) genes demonstrated a distinct evolutionary trajectory for the representative strain JH-2-11, which diverged significantly from the other three isolates (JH-1-1, JH-89, and JH-91). Specifically, the M gene of JH-2-11 was more closely related to an H11N3 isolate from Bangladesh and an H5N7 isolate from Dongting Lake. Furthermore, its NS gene clustered in a separate evolutionary branch alongside an H4N6 isolate from wild birds in Guangdong and an H10N3 isolate from Mongolia. These data provide compelling evidence that, although JH-2-11 was isolated from the same geographical region (Jianhu area) as the other H6N2 strains, its M and NS segments were acquired through completely independent reassortment events.

Further, we analyzed the mutations in the key amino acid sites of the eight internal gene segments (Table 3). The PB2 segment-encoded protein showed mutations at the L89V site (which increases virus pathogenicity in mice) but no mutations at the E627K or D701N sites. The PB1-encoded protein contained mutations at L473V and L13P (associated with viral replication and increased pathogenicity in mammals), whereas the PA-encoded protein contained mutations at N383D and S515T. The NS segment-encoded protein had a mutation at site P42S, whereas the M1-encoded protein had mutations at N30D, I43M, and T215A. These mutations may enhance the replication capability and pathogenicity of H6N2 isolates in various hosts, including mice.

### 3.4. In Vitro Replication Assay and Growth Kinetics H6N2 Subtype JH-2-11 Isolate

**Figure 4:**
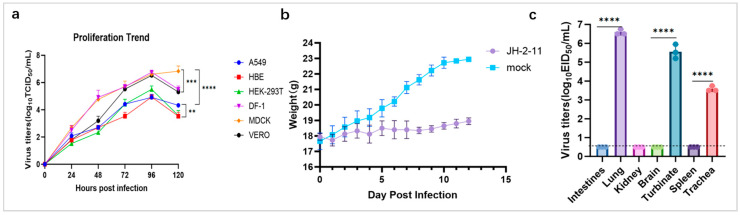
Growth kinetics of AIV subtype JH-2-11 isolate in human and animal-derived cell lines, changes in body weight, and virus titration in different organs of BALB/c mice. (**a**) Growth kinetics of duck-origin JH-2-11 isolate in human and animal-derived cell lines. (**b**) Changes in the body weight of mice after viral infection. (**c**) Virus titration in the organs of BALB/c mice at 3 dpi. The data shown are the means of three replicates, and the error bars indicate standard deviations. ** (*p* < 0.01), *** (*p* < 0.001), **** (*p* < 0.0001).

To better understand the replication and growth kinetics of the duck-origin JH-2-11 AIV, we infected all human and animal-derived cell lines, such as HBE, HEK-293T, A549, MDCK, VERO, and DF-1, with a virus dose of MOI = 0.01, and the supernatants were collected at 24, 48, 72, 96, and 120 hpi to determine the virus titer and assess virus replication ability. The results demonstrated that the JH-2-11 isolate exhibited highly robust replication efficiency in MDCK, DF-1, and VERO cells. In these three highly susceptible cell lines, viral titers increased rapidly starting from 24 h post-infection (hpi) and peaked at 96 hpi. Notably, MDCK cells maintained the highest viral yield at 120 hpi without exhibiting a significant decline. In contrast, A549, HEK-293T, and HBE cells exhibited a relatively weaker capacity to support the proliferation of this strain. At 96 hpi, the peak titers in these three cell lines were significantly lower than those in the aforementioned highly susceptible cells (by a difference of approximately 1.5 to 2.0 logs). Among them, HBE cells displayed the lowest overall replication efficiency, with a peak titer of less than 5.0 log_10_ TCID_50_/mL. Following 96 hpi, viral titers in most cell lines, except for MDCK, decreased to varying extents (Figure 4a).

### 3.5. In Vivo Replication of H6N2 Subtype JH-2-11 Isolate

BALB/c mice infected with the duck-derived H6N2 strain JH-2-11 showed no obvious clinical symptoms within 12 days post-infection (dpi). Compared with the negative control group, BALB/c mice infected with the JH-2-11 strain exhibited a slower weight gain during the infection period. The body weight of infected mice remained at approximately 18 g from 0 to 12 days post-infection without a significant increase, whereas mice in the negative control group showed a gradual weight increase from 18 g to 22 g (Figure 4b). The organ titration results showed that lung, nasal turbinate, and trachea tissues were all positive in embryonated egg inoculation, while all other tissues and organs were negative. The lung tissue exhibited the highest EID_50_ of up to 6.5 log_10_ EID_50_/mL, indicating that the JH-2-11 strain was capable of infecting mice and transmitting through the respiratory tract (Figure 4c).

**Figure 5:**
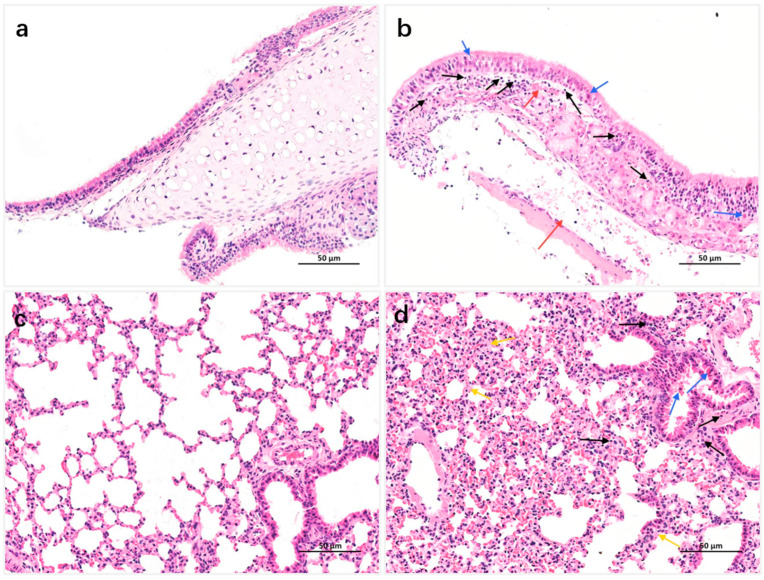
H&E staining results of the nasal turbinate and lung tissues of mice before and after infection with the JH-2-11 AIV strain. (**a**) Negative control group of nasal turbinate tissue, (**b**) Pathological changes in nasal turbinate tissue in mice infected with the JH-2-11 AIV strain, (**c**) negative control group of lung tissue, (**d**) Pathological changes in the lung of JH-2-11 infected mice.

H&E staining results of nasal turbinate and lung tissues from mice infected with the JH-2-11 strain on day 3 post-infection are shown in Figure 5. In the negative control group, the nasal turbinate microstructure was essentially normal, with regularly arranged ciliated columnar epithelium and no definite inflammatory cell infiltration outside the blood vessels (Figure 5a). In the experimental group, the respiratory epithelial cells of the nasal turbinate were largely normal, with a small amount of pyknotic degeneration observed in the ciliated columnar epithelium (blue ↑), and the overall arrangement was relatively regular. Moderate inflammatory cell infiltration was observed in the lamina propria (black ↑), and widespread vascular congestion was noted (red ↑) (Figure 5b). In the negative control group, the lung parenchymal structure was essentially normal; no necrosis or desquamation of epithelial cells was observed in the bronchi at all levels, nor was there interstitial inflammatory cell infiltration, edema, alveolar wall thickening, or obvious hyperplasia of alveolar cells (Figure 5c). In the experimental group, extensive atelectasis was observed in the lung tissue. Sparse inflammatory cell infiltration was present around the bronchi (black ↑); the bronchial epithelial cells within the lumen were disorganized, with necrosis and desquamation evident (blue ↑). Focal thickening of the alveolar septa was also present (orange ↑) (Figure 5d).

**Figure 6:**
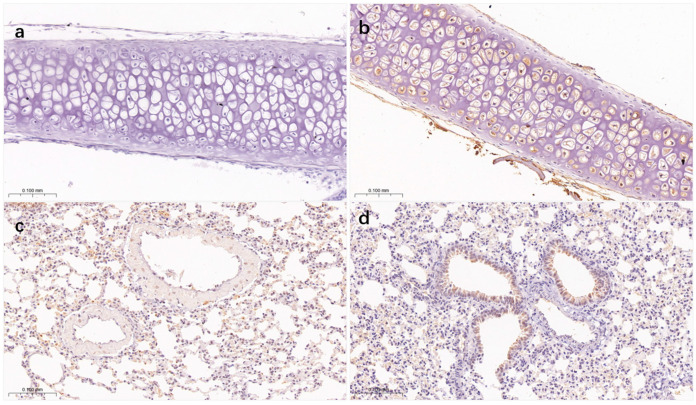
IHC staining was performed to detect the viral NP protein: (**a**) negative control group of nasal turbinate tissue, (**b**) IHC images of nasal turbinate tissues from mice infected with the JH-2-11 strain, (**c**) negative control group of lung tissue, (**d**) IHC images of lung tissues from mice infected with the JH-2-11 strain.

Immunohistochemistry results revealed positive staining for influenza virus NP antigen in both nasal turbinate and alveolar tissues of mice. In the negative control group, no obvious NP protein staining (brown signal) was observed in the mucosal epithelium or chondrocytes of the nasal turbinate, and the tissue architecture remained intact with no signs of viral infection (Figure 6a). In the experimental group, abundant and distinct brownish NP antigen-positive signals were detected in the cytoplasm of mucosal epithelial cells and chondrocytes in the nasal turbinate, with widespread signal distribution (Figure 6b), indicating that the JH-2-11 strain achieved effective replication in the nasal turbinate by 3 days post-infection, with abundant viral antigen expression. In the negative control group, only minimal faint staining was observed in alveolar epithelial cells, bronchial epithelial cells, and lung interstitium, which was considered within the background signal range (Figure 6c). In contrast, strong positive brownish staining was detected in the cytoplasm of bronchial epithelial cells in the experimental group, along with positive signals in the alveolar septa and some alveolar epithelial cells (Figure 6d). These findings demonstrate that the virus not only replicated extensively in bronchial epithelial cells but also had disseminated into the alveolar region, causing widespread infection throughout the lung tissue.

## 4. Discussion

The geographical location of Yunnan Province, situated at the intersection of the Central Asian and East Asian-Australasian migratory flyways, creates a unique ecological nexus that facilitates complex inter-subtype reassortment of avian influenza viruses (AIVs). In this study, while H6 subtype viruses are classically recognized as low pathogenic avian influenza (LPAI) viruses in poultry, their threat to public health cannot be understated, as evidenced by the first human infection with a novel reassortant H6N1 virus in Taiwan in 2013 [20]. In this study, we characterized four H6N2 isolates (JH-89, JH-91, JH-1-1, and JH-2-11) from wild and domestic birds in the Jianhu Lake region. The genomic and phenotypic analyses reveal that while these viruses maintain typical LPAI characteristics, they possess unique reassortment patterns and molecular markers that suggest a latent potential for mammalian adaptation.

The genomic composition of the identified H6N2 strains reflects a highly mosaic evolutionary history. While the surface glycoproteins (HA and NA) and the majority of internal genes (PB2, PB1, PA, and NP) of the four isolates share a common Eurasian lineage background with viruses circulating in waterfowl, the isolate JH-2-11 exhibits a distinct evolutionary trajectory. Unlike the other three strains, the M and NS segments of JH-2-11 originated from independent sources, with the NS segment closely clustering with H10N3-like viruses. This topological incongruence highlights the role of H6 viruses as an active genetic mixing vessel in wetland ecosystems, where co-circulation with diverse subtypes provides a constant supply of internal gene modules. Despite these diverse origins, the maintenance of high sequence conservation in the ribonucleoprotein (RNP) complex across all four isolates underscores the strict functional constraints on the viral replication machinery in avian populations.

Molecular analysis of the receptor-binding site (RBS) revealed that all four isolates retain the avian-preferred residues Q226 and G228 (H3 numbering), consistent with their isolation from aquatic birds. Although classical mammalian-adaptive mutations such as PB2-E627K or D701N were absent, the emergence of PB2-L89V and NS1-P42S in JH-2-11 is particularly noteworthy. Previous experimental evidence has demonstrated that the PB2-L89V substitution can enhance polymerase activity in mammalian cells [14,21] while the NS1-P42S mutation is strongly associated with increased antagonism of the host interferon response and enhanced virulence [22]. It is highly plausible that the acquisition of the distinct NS segment harboring P42S synergizes with polymerase mutations like L89V to overcome host restriction factors. This accumulation of non-canonical markers, alongside mutations in PB1 (L473V) and M1 (N30D), suggests a polygenic adaptation process. This stepping-stone evolution could allow the virus to partially bypass the strict requirement for E627K, especially when circulating in complex ecological interfaces such as live-bird markets [23].

The functional impact of these genetic features was directly reflected in vitro replication assays. JH-2-11 demonstrated broad cellular permissiveness, replicating efficiently in avian (DF-1), canine (MDCK), and simian (VERO) cells. More importantly, the virus achieved productive replication in human respiratory cell lines (A549 and HBE), albeit with lower peak titers compared to MDCK cells. This moderate replication efficiency in A549 cells, despite a strict avian-type receptor preference, indicates that the unique internal gene of JH-2-11—particularly its reassortant M and NS segments—may provide sufficient metabolic fitness to support viral transcription in human cells. This aligns with a phenotype of “potential for cross-species infection” rather than an immediate pandemic threat, necessitating the continued monitoring of these “pre-adapted” internal gene cassettes It is important to note a methodological limitation in vitro replication assays: viral titers from all cell lines were exclusively determined using the TCID_50_ assay on MDCK cells.

The in vivo pathogenicity data further temper the zoonotic risk assessment while precisely defining the virus’s respiratory tropism. Infected BALB/c mice remained asymptomatic with no mortality, confirming the inherent low pathogenic nature of JH-2-11 in a mammalian model. However, even without prior mammalian adaptation, the virus achieved high titers (6.5 EID_50_/mL) in the lungs and nasal turbinates, accompanied by visible pathological changes. H&E staining provided direct evidence of viral replication in the bronchial epithelium and subsequent inflammatory cell infiltration. The robust replication restricted solely to the respiratory tract, coupled with the lack of systemic spread, suggests an “incomplete adaptation” state: while the physical species barrier for entry is partially permeable, systemic dissemination is currently limited by the absence of pivotal markers like PB2-627K [24].

## 5. Conclusions

In conclusion, this study identifies a novel H6N2 reassortment in Southwest China with a unique internal gene configuration. While the current isolates do not possess the molecular or phenotypic traits for sustained human-to-human transmission, their ability to replicate in human respiratory cells and cause localized infection in mice underscores their evolutionary flexibility. The limitations of this study, including the focus on a single representative isolate for animal trials and the probabilistic nature of genotypic inferences, suggest that broader surveillance is required. Because progeny virions derived from different host cells may exhibit varying receptor-binding affinities or infectivity profiles, titrating yields from human or avian cell substrates exclusively on MDCK cells might differentially underestimate the actual viral yield from those non-MDCK lines. Future efforts should prioritize integrated genotypic and phenotypic monitoring at migratory intersections to detect early signals of H6-derived viruses moving toward human-type receptor specificity or enhanced mammalian virulence.

## Figures and Tables

**Table 1:** Homology analysis of each gene segment of the H6N2 isolates.

Virus	Genes	Virus Strains	Host Species	Homology (%)	Accession No.
JH-89	PB2	A/environment/Japan/KU-4h/2021(H3N8)	-	99.38	OR044155.1
	PB1	A/wild duck/Guangdong/ZJ1686/2023(H4N6)	wild duck	99.03	PQ395388.1
	PA	A/Spot-billed duck/South Korea/KNU2022-73/2022(H4N6)	Spot-billed duck	99.26	PP746223.1
	HA	A/duck/Kaohsiung/19WB0234-29/2019(H6N2)	duck	98.78	OR949620.1
	NP	A/mallard duck/Bangladesh/59338/2023(H10N7)	mallard duck	99.20	PP883010.1
	NA	A/duck/Bangladesh/58751/2023(H6N2)	duck	98.30	PP680422.1
	M	A/duck/Bangladesh/41797/2019(H3N8)	duck	99.59	MW466354.1
	NS	A/Whooper_Swan/South Korea/segment8/2023(H5N6)	Whooper_Swan	99.64	PP348173.1
JH-91	PB2	A/environment/Japan/KU-4h/2021(H3N8)	-	99.43	OR044155.1
	PB1	A/wild duck/Guangdong/ZJ1686/2023(H4N6)	wild duck	99.03	PQ395388.1
	PA	A/Spot-billed duck/South Korea/KNU2022-73/2022(H4N6)	Spot-billed duck	99.30	PP746223.1
	HA	A/duck/Kaohsiung/19WB0234-29/2019(H6N2)	duck	98.67	OR949620.1
	NP	A/mallard duck/Bangladesh/59338/2023(H10N7)	mallard duck	99.20	PP883010.1
	NA	A/duck/Bangladesh/58751/2023(H6N2)	duck	98.30	PP680422.1
	M	A/duck/Bangladesh/41797/2019(H3N8)	duck	99.59	MW466354.1
	NS	A/Whooper_Swan/South Korea/segment8/2023(H5N6)	Whooper_Swan	99.64	PP348173.1
JH-1-1	PB2	A/environment/Japan/KU-4h/2021(H3N8)	-	99.38	OR044155.1
	PB1	A/wild duck/Guangdong/ZJ1686/2023(H4N6)	wild duck	99.03	PQ395388.1
	PA	A/Spot-billed duck/South Korea/KNU2022-73/2022(H4N6)	Spot-billed duck	99.26	PP746223.1
	HA	A/duck/Kaohsiung/19WB0234-29/2019(H6N2)	duck	98.78	OR949620.1
	NP	A/mallard duck/Bangladesh/59338/2023(H10N7)	mallard duck	99.20	PP883010.1
	NA	A/duck/Bangladesh/58751/2023(H6N2)	duck	98.30	PP680422.1
	M	A/duck/Bangladesh/41797/2019(H3N8)	duck	99.59	MW466354.1
	NS	A/Whooper_Swan/South Korea/segment8/2023(H5N6)	Whooper_Swan	99.64	PP348173.1
JH-2-11	PB2	A/environment/Japan/KU-4h/2021(H3N8)	-	99.39	OR044155.1
	PB1	A/wild duck/Guangdong/ZJ1686/2023(H4N6)	wild duck	99.03	PQ395388.1
	PA	A/Spot-billed duck/South Korea/KNU2022-73/2022(H4N6)	Spot-billed duck	99.26	PP746223.1
	HA	A/duck/Kaohsiung/19WB0234-29/2019(H6N2)	duck	98.67	OR949620.1
	NP	A/mallard duck/Bangladesh/59338/2023(H10N7)	mallard duck	99.20	PP883010.1
	NA	A/duck/Bangladesh/58751/2023(H6N2)	duck	98.30	PP680422.1
	M	A/duck/Bangladesh/38827/2019(H11N3)	duck	98.87	MT090539.1
	NS	A/duck/Mongolia/518/2015(H10N3)	duck	99.05	LC121392.1

**Table 2:** Analysis of protein glycosylation sites and resistance mutations in HA and NA.

**H6N2 Isolates**	**Cleavage Site**	**HA Protein Glycosylation Site**																														**Amino Acid Mutations**					
		**27**					**39**					**182**					**306**					**489**					**557**					**Q226L**			**G228S**		
JH-89	PQIENR↓GLF	NST					NVT					NNT					NKT					NGT					NGS					Q			G		
JH-91	PQIENR↓GLF	NST					NVT					NNT					NKT					NGT					NGS					Q			G		
JH-1-1	PQIENR↓GLF	NST					NVT					NNT					NKT					NGT					NGS					Q			G		
JH-2-11	PQIENR↓GLF	NST					NVT					NNT					NKT					NGT					NGS					Q			G		
**H6N2 Isolates**	**NA Deletion**	**NA Protein Glycosylation Site**																														**Amino Acid Mutations**					
		**61**						**69**						**86**						**146**						**234**						**E119G**		**Q136L**		**R292K**	
JH-89	No	NIT						NNT						NWS						NGT						NGT						E		Q		R	
JH-91	No	NIT						NNT						NWS						NGT						NGT						E		Q		R	
JH-1-1	No	NIT						NNT						NWS						NGT						NGT						E		Q		R	
JH-2-11	No	NIT						NNT						NWS						NGT						NGT						E		Q		R	

**Table 3:** Analysis of mutations at key amino acid sites in the eight internal genetic segments.

Gene	Site	Function	JH-89	JH-91	JH-1-1	JH-2-11
HA	Q226L	Enhance the pathogenic potential of viral infection in mammals	Q	Q	Q	Q
	G228S		G	G	G	G
NA	E119G	NA inhibitor resistance	E	E	E	E
	Q136L		Q	Q	Q	Q
	R292K		R	R	R	R
M1	N30D	Improve the adaptability of the virus in mammalian cells	D	D	D	D
	T215A		A	A	A	A
	I43M		M	M	M	M
NS	P42S	Viruses are more pathogenic in mammalian hosts	S	S	S	A
NP	I353V	Enhance the genomic stability of the virus within the host cell	V	V	V	V
	N319K		N	N	N	N
PB1	L473V	Enhanced the replication ability of the viral polymerase in mammals and the pathogenicity of the virus to mammals	V	V	V	V
	L13P		P	P	P	P
PA	N383D	Enhance the replication ability of the virus or increase its pathogenicity to the host.	D	D	D	D
	S515T		T	T	T	T
PB2	L89V	Increase the pathogenicity of mice	V	V	V	V
	E627K	Increase the ability of transmission among mammals	E	E	E	E
	D701N		D	D	D	D

## Data Availability

The data analyzed in this study have been submitted to the GenBank of the NCBI database under accession numbers mentioned below: A/Areda cinerea/Yunnan/JH-89/2024 (H6N2): JH-89 (PB2: PZ177692, PB1: PZ177696, PA: PZ177676, HA: PZ177668, NP: PZ177684, NA: PZ177672, M: PZ177688, NS: PZ177680). A/Areda cinerea/Yunnan/JH-91/2024 (H6N2): JH-91 (PB2: PZ177693, PB1: PZ177697, PA: PZ177677, HA: PZ177669, NP: PZ177685, NA: PZ177673, M: PZ177689, NS: PZ177681). A/Duck/Yunnan/JH-1-1/2025(H6N2): JH-1-1 (PB2: PZ177694, PB1: PZ177698, PA: PZ177678, HA: PZ177670, NP: PP PZ177686, NA: PZ177674, M: PZ177690, NS: PZ177682). A/Mareca penelope/Yunnan/JH-2-11/2025(H6N2): JH-2-11 (PB2: PZ177695: JH-91 (PB1: PZ177699, PA: PZ177679, HA: PZ177671, NP: PZ177687, NA: PZ177675, M: PZ177691, NS: PZ177683).

## Supplementary Materials

The following supporting information can be downloaded at: https://www.mdpi.com/article/10.3390/microorganisms14040895/s1. Table S1. Sample sources and AIV numbers from different locations. Table S2. AIV isolate names and their concentrations. Table S3. Primer sequences used for the Pcr detection and amplification.
