# Supplementary material for: Characterization and Genetic Evolution of H6N2 Subtype AIV Isolates from Aquatic Birds
Source: Microorganisms. 2026 Apr 16;14(4):895. doi: 10.3390/microorganisms14040895 (PMC13118982; doi:10.3390/microorganisms14040895)
Supplement: Supplementary file 1 [file microorganisms-14-00895-s001.zip › microorganisms-4184425-supplementary.pdf]

Supplementary Table S1. Sample sources and AIV numbers from different locations.

Host species	number	source	Location	AIV number	H6N2 number
Grey heron	97	feces	China:Jianhu	5	2
Eurasian wigeon	51	feces	China:Jianhu	1	1
Duck	53	feces	China:Jianhu	6	1
Ruddy shelduck	67	feces	China:Jianhu	0	0
Purple swamphen	78	feces	China:Jianhu	1	0
Chicken	40	feces	China:Jianhu	0	0
Mallard	32	feces	China:Jianhu	0	0
Night heron	36	feces	China:Jianhu	0	0
Egret	25	feces	China:Jianhu	0	0
Coot	32	feces	China:Jianhu	0	0
Bar headed goose	56	feces	China:Jianhu	0	0
Grey goose	52	feces	China:Jianhu	1	0

Supplementary Table S2. AIV isolate names and their concentrations.

Isolates name	Allantoic fluid (copies/μL)	HA titer	EID_{50} ($lgEID_{50}$/ml)	$TCID_{50}$ ($lgTID_{50}$/ml)
JH-89	3.98×10^{6}	$1:2^{8}$	6.5	5.167
JH-91	3.76×10^{5}	$1:2^{7}$	5.75	3.5
JH-1-1	3.49×10^{5}	$1:2^{7}$	5.5	3.75
JH-2-11	4.2×10^{7}	$1:2^{9}$	7.167	6.25

Supplementary Table S3. Primer sequences used for the Pcr detection and amplification.

**Primer name**	**Primer sequence (5′-3′)**
H6-F-1	AGCRAAAGCAGGGGAAAATGATTG
H6-R-1165	TCTTTGTCTGCTGCATATCCTGA
PB2-F-128	CTGCCCTYAGGATGAARTGGATG
PB2-R-1137	YCTGGTTGCTTTCCTTAGAATGG
PB2-F-738	GGGAGGGGAGGTAAGRAATGATG
PB2-R-1520	TGATCTCGGACTCTCAAGAAACG
PB2-F-1561	ACACAGGGAACAGARAAACTRAC
PB2-R-2113	CRTATCTTTTGTCTTCTTTGCCCAG
PB2-F-1934	TGAGAAMCCTTGTGAGAGGCAAC
PB2-R-2284	GACACTAWWTGATGGCCATCCG
NP-F-346	ATCAGGAGAATCTGGCGTCAAG

NP-R-725	ACTTGGTCCATCATTGCTCTTTG
PA-F-468	CACCAAGGCGGACTACACCC
PA-R-1141	AGTCCACTTTTTCTGGTGCCATG
PA-F-1269	TGAACTTGATGAAATAGGGGAAGAC
PA-R-1709	GTCCCATTGGTTCTCACATACAG
PB1-F-53	TAAGTACTACATTCCCTTACACTGG
PB1-R-1141	AATATTTCAGATCAATGTTTGCAAG
PB1-F-555	CCAGAGAAAGAGAAGAGTRAGGG
PB1-R-1032	GAACATTATAGGGGCAATGCTCARG
PB1-F-998	TTAGAAATGTCYTGAGCATTGCC
PB1-R-1499	CCATAGCGGTAGAAAAAGCTTGTG
PB1-F-1420	GGGATCAACATGAGCAAAAAGAAGTC
PB1-R-1939	GCATTACCACAGCATTGTTTACGG
PB1-F-1845	GATGGATGAGGACTACCAGGGC
PB1-R-2282	TAAATTCACTATTTTTGCCGTCTGAGC
